# An Objective Functional Characterisation of Head Movement Impairment in Individuals with Neck Muscle Weakness Due to Amyotrophic Lateral Sclerosis

**DOI:** 10.1371/journal.pone.0169019

**Published:** 2017-01-09

**Authors:** Silvia Pancani, Wendy Tindale, Pamela J. Shaw, Christopher J. McDermott, Claudia Mazzà

**Affiliations:** 1 Department of Mechanical Engineering, University of Sheffield, Sheffield, United Kingdom; 2 INSIGNEO Institute for in silico Medicine, University of Sheffield, Sheffield, United Kingdom; 3 NIHR Devices for Dignity Healthcare Technology Co-operative, Sheffield Teaching Hospitals NHS Foundation Trust, Royal Hallamshire Hospital, Sheffield, United Kingdom; 4 Sheffield Teaching Hospitals NHS Foundation Trust, Sheffield, United Kingdom; 5 Sheffield Institute for Translational Neuroscience, University of Sheffield, Sheffield, United Kingdom; Tokai University, JAPAN

## Abstract

**Background:**

Neck muscle weakness and head drop are well recognised in patients with Amyotrophic lateral sclerosis (ALS), but an objective characterisation of the consequent head movement impairment is lacking. The aim of this study was to quantitatively characterise head movements in ALS compared to aged matched controls.

**Methods:**

We evaluated two groups, one of thirteen patients with ALS and one of thirteen age-matched controls, during the execution of a series of controlled head movements, performed while wearing two inertial sensors attached on the forehead and sternum, respectively. We quantified the differences between the two groups from the sensor data using indices of velocity, smoothness and movement coupling (intended as a measure of undesired out of plane movements).

**Findings:**

Results confirmed a general limitation in the ability of the ALS patients to perform and control head movements. High inter-patient variability was observed due to a wide range of observed functional impairment levels. The ability to extend the head backward and flex it laterally were the most compromised, with significantly lower angular velocity (*P* < 0.05, Cohen’s d > 0.8), reduced smoothness and greater presence of coupled movements with respect to the controls. A significant reduction of angular velocity (P < 0.05, Cohen’s d > 0.8) in extension, axial rotation and lateral flexion was observed when patients were asked to perform the movements as fast as possible.

**Interpretation:**

This pilot study is the first study providing a functional objective quantification of head movements in ALS. Further work involving different body areas and correlation with existing methods of evaluating neuromuscular function, such as dynamometry and EMG, is needed to explore the use of this approach as a marker of disease progression in ALS.

## Introduction

Amyotrophic lateral sclerosis (ALS), also known as motor neurone disease is a degenerative disease primarily of motor neurones that leads to progressive muscle weakness. The consequences in terms of motor function differ depending on the extent to which upper and lower motor neurons are affected by the degeneration [1]. The onset of ALS tends to be focal with weakness presenting in a particular group of muscles first. This is usually distally in one limb with spread to other muscles within this limb and beyond over time. Bulbar and respiratory muscles are also affected, as are the muscles in the neck which support the head and enable its motion. Muscle weakness in the neck usually affects the neck extensor muscles, with or without the involvement of the neck flexors [2]. In those cases, a consequent head drop exacerbates problems with swallowing, communicating and breathing, causing significant disability and difficulties in social interactions. It has been reported that in ALS patients head drop affects quality of life [3] and, in order to improve their posture and overcome those difficulties, patients are advised to wear a cervical orthosis [2, 3]. It has also been recently shown that neck muscle weakness leads to an increasing difficulty in performing the activities of daily living (ADL) and is negatively associated with survival time in ALS patients [4].

A quantification of the interaction between neck muscle weakness due to ALS and consequent functional limitation, to the authors’ knowledge, has only been performed by testing muscle weakness with manual muscle testing and by assessing the ability to perform ADL using a clinical scale [4]. The main limitations of both functional rating scales and manual muscle testing (MMT) are that they are evaluator-dependent, provide ordinal data, which may lack sensitivity in the presence of small changes, and that they provide steps between grades which are not guaranteed to be qualitatively equivalent for each interval [5]. This can cause the disease to progress for an extended period before it is detected by a change in the MMT score. Furthermore, it has been proposed that there can be some critical levels at which a small decline in strength leads to a large functional loss [5]. Additional instruments, such as hand-held dynamometers (HHD), have been introduced in clinical practice to obtain a quantitative and more accurate assessment. These devices are portable, easy to use and relatively inexpensive. Nevertheless, they evaluate only isolated strength over specific muscle groups and do not provide an assessment of the overall function of a joint [6]. Thus, an assessment based only on muscle strength evaluation conveys limited information with regard the actual level of progression of the disease and there is a need for the development of tools that enable a more function based objective quantitative assessment of execution of movement. In the specific case of the assessment of functional neck impairment in ALS patients, since currently used ALS clinical scales do not take into account any measure of neck function and MMT and HHD techniques usually only evaluate neck extensor muscles, there is a need for a better outcome measure.

In an attempt to investigate the ability of different cohorts of participants to perform head movements, a number of researchers have measured both the velocity and the smoothness of these movements [7–10]. The reduction of the velocity of head movements has been demonstrated to be a feature characteristic of individuals with chronic neck pain [7] and a marker of neck pathologies, such as cervical dystonia [8]. The fluidity, or smoothness, of a movement is often used as an indicator of unimpaired movement control and coordination. Several studies, in fact, have recognised that a lack of coordination, due to advanced age [9] or pathological conditions is typically associated with reduced smoothness [10]. Since impaired coordination and poor muscle control are primary consequences of altered muscle strength [11], it is reasonable to hypothesise that the assessment of movement smoothness, together with the measurement of velocity parameters, could allow the quantitative evaluation of a patient’s ability to perform head movements and provide valuable information to inform clinical care.

An additional feature of potential interest for a quantitative assessment of specific residual abilities is the so-called coupling of the movements [12]. Pure head flexion-extension, axial-rotation and lateral flexion are movements executed in the sagittal, transverse and frontal planes, respectively. However, whilst the orientation of the cervical vertebral bodies allow for pure flexion-extension, they impede pure lateral flexion, and a simultaneous axial rotation is typically observed [12]. The out of plane movement resulting from this combination is often described as a coupling of the primary (lateral flexion) and the secondary (axial rotation) movements. Similar physiological movement couplings can, of course, also occur in other planes and for the other movements. Coupled mechanisms of the upper cervical spine have been shown to be significantly increased in pathological conditions such as cervical dystonia, likely due to the co-contraction of the cervical muscles which is known to occur in this condition [8] and with increasing age [12]. In patients with ALS, the presence of increased coupled movements of the neck could be expected as a result of neighbouring muscles being employed to compensate for muscle weakness. Its quantification may hence add useful information for the functional assessment of these patients. Coupled movements of the neck were investigated through the assessment of the movements of the head with respect to the trunk.

The aim of this pilot study was to quantitatively characterise head movements with regard to velocity, smoothness and coupling in ALS compared to aged matched controls.

## Methods

### Participants

A cohort of thirteen individuals affected by neck muscle weakness due to ALS (6 females, 7 males, age range 45–74 years) participated in the study. The severity of the disease was assessed in patients by using the ALS Functional Rating Scale-Revised (ALSFRS-R). The ALSFRS-R is a validated ordinal scale, commonly used in clinics to estimate a patient’s degree of functional impairment [13]. The scale ranges from 0 (worst) to 48 (best). The participating patients’ characteristics (age, ALSFRS-R score at the time of recording and time course from diagnosis to recording) are summarized in Table 1. Inclusion criteria were: ability to understand instructions and give informed consent, definite diagnosis of ALS accordingly to the modified El Escorial criteria [14]; absence of comorbidities and presence of neck muscle weakness, as observed by a physician, as well as the presence of residual muscle strength to enable the performance of the test procedure. Individuals that were not able to lift their head at all from their chest were excluded from the study. Thirteen age-matched healthy individuals (6 females, 7 males, age range 44–75 years) were also enrolled. Inclusion criteria for healthy individuals were: the absence of symptoms or history of cervical spine disorders. All the participants were informed about the protocol through an information sheet and signed a consent form prior to their inclusion in the study. The study was reviewed and approved by the NRES Committee North East- Newcastle and North Tyneside (REC project number STH18733).

**Table 1:** Patients’ characteristics. y = yes, n = no.

Participant	Age (years)	ALSFRS-R score (0–48)	Time from diagnosis (months)	Movements Max Amplitude	Movements Max Speed
1	69	30	11.5	y	y
2	74	13	49	y	n
3	69	44	1.5	y	y
4	63	18	36.5	y	y
5	58	43	34.5	y	y
6	53	22	2.5	y	n
7	69	23	18	y	n
8	53	34	10	y	y
9	65	19	36	y	n
10	74	17	59	y	y
11	50	23	57	y	y
12	45	18	36	y	y
13	63	36	45	y	y

### Protocol

**Fig 1:**
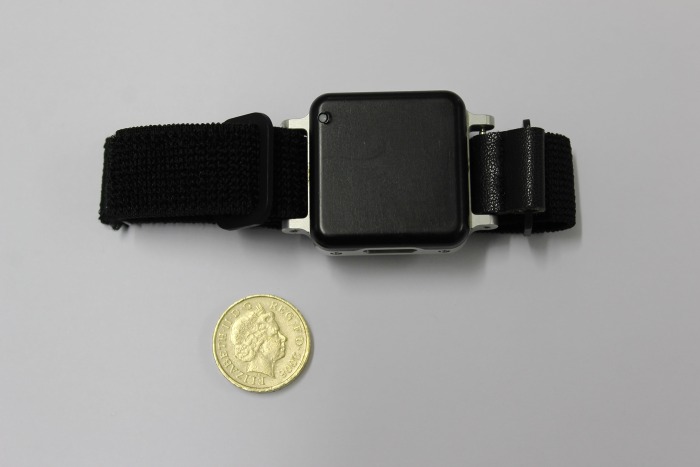
Inertial Magneto Unit (IMU).

The assessment was performed using two inertial magneto units (IMUs) (OPAL, APDM Inc., USA, sampling frequency 128 samples/s, Fig 1), following a previously adopted protocol [15]. Each IMU is equipped with a tri-axial accelerometer, a tri-axial gyroscope and a tri-axial magnetometer to measure linear acceleration, angular velocity and orientation. The two IMUs were firmly attached on the forehead and sternum of each participant using double sided tape. A functional calibration approach (described below) was adopted. The sensor attached to the forehead was used to record the movements of the head while the sensor attached to the sternum was used to detect undesired movements of the trunk.

Each participant was asked to sit on a chair and perform a series of active head movements: flexion (F), extension (E), axial rotation (AR, toward their left and right side) and lateral flexion (LF, toward their left and right side), starting from their own neutral position (NP) and looking ahead. To make sure they were well familiarized with the protocol, all participants were asked to practice the sequence of movements before the acquisition session started. All movements were performed first reaching the maximum amplitude (i.e. the participants were asked to move their head as far as possible from the neutral position), then at maximum speed (i.e. the participants were asked to move their head as fast as possible). The first condition was used to evaluate participants’ ability to perform head movements while they endeavoured to reach their maximum range of motion in each direction, but still in a controlled state. The second condition was used to evaluate participants’ ability to perform head movements in a less controlled state. Each movement was repeated three times. The participants with ALS were asked to perform the movements at maximum speed only if they felt comfortable with doing it. The assessment took approximately 30 minutes.

### Data Processing

**Fig 2:**
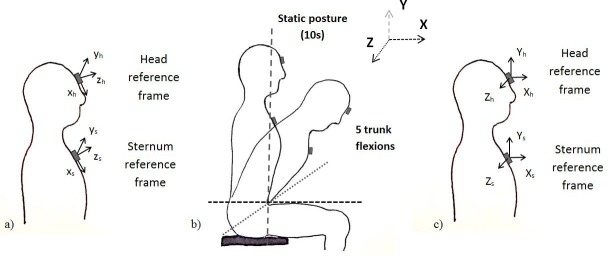
Functional calibration procedure. a) Initial orientation of the sensors’ frame; b) functional calibration tasks; c) Final orientation of the sensors’ frame according to the reference frame built through the functional calibration tasks.

Data were analysed using custom procedures written in MATLAB (Mathworks Inc., Natick, MA, USA). Prior to the analysis, signals were filtered using a 4th order zero-lag Butterworth filter. The cut-off frequency value was then conservatively set to 10 Hz, after having checked the frequency content of signals collected. Thereafter, IMUs orientation was estimated in order to align the sensor placed on the forehead with the sensor placed on the sternum and remove orientation errors that could occur due to their placement. A functional calibration approach, described in detail in previous studies [15, 16], was used for this purpose. The accelerations and angular velocities of forehead and sternum were expressed according to a common anatomical reference frame, which was defined using a functional calibration task. The task consisted of asking participants, while sitting on a chair, to look forward for ten seconds and then perform five trunk flexions. Once the two sensor reference frames were aligned to the anatomical reference frame, the accelerations and the velocities recorded at the sternum level were subtracted from those recorded at the head level. This enabled movements of the head arising from a movement of the trunk to be isolated and removed [17]. The location of the IMUs on the participants’ forehead and sternum and the functional procedure used to align their reference frames are described in Fig 2.

**Fig 3:**
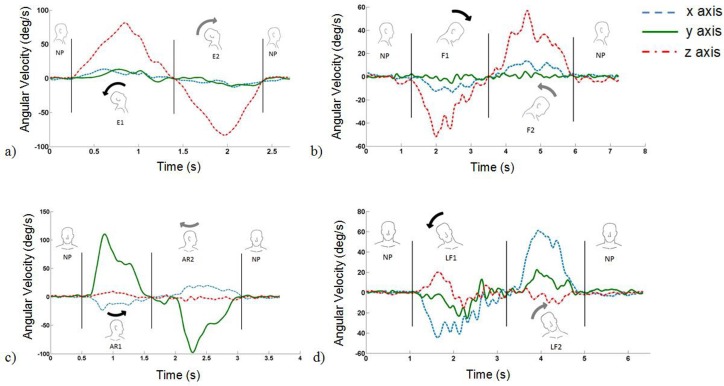
Exemplifying angular velocity graphs as measured during head movements in one patient with ALS. a) Head extension: the movement starts from the neutral position (NP), then the head is moved backward (E1), from the NP until the neck is fully stretched, and finally forward (E2), back to the initial NP. Corresponding graphs for other movements: b) flexion, c) axial rotation, d) lateral flexion.

Every movement (M) was sub-divided in two phases: movement away from neutral position (M1) and movement back to neutral position (M2), as they involve different groups of muscles. For each movement the end of the first phase was detected when the angular velocity first crossed the zero (from positive to negative or vice versa, according to the movement), which coincided with the moment when the direction of the movement is reversed (Fig 3).

The following parameters were used to quantify the head movements: mean angular velocity (ω_m_), peak angular velocity (ω_p_), normalized jerk (NJ), and ratio of movement coupling (RMC). ω_m_ and ω_p_ were calculated using the signal recorded by the tri-axial gyroscope. To calculate ω_m_ the signal was averaged through the duration of the movement while, to evaluate ω_p,_ its peak value was considered.

The jerk (J) was initially computed as the first time derivative of the linear acceleration measured by the tri-axial accelerometer. Then, a time-integrated squared jerk was calculated and normalized with respect to the mean absolute acceleration and duration of the movement, using the following equation [18]:

$$NJ=\sqrt{\frac{1}{2}\frac{T}{{\bar{a}}^{2}}\int J^{2}(t)dt},$$ (1)

where T is the duration of the movement and $\bar{a}$ is its mean absolute acceleration. By definition, lower values of NJ are associated with smoother movements [18].

As described in the introduction, a pure primary movement would entail a rotation in only one of the three main anatomical planes. In this case, the direction of the angular velocity would coincide with the direction of the main anatomical axis perpendicular to the anatomical plane in which the movement is performed and the relevant angular velocity signal would be the highest among the three recorded ones. The presence and amount of coupled movements, on the contrary, entails higher values also of the other angular velocity components. The presence of coupled movements was hence quantified using the following ratio:

$$RMC=\frac{A_{j}+A_{k}}{A_{i}},$$ (2)

where *i* is the axis around which the primary movement is performed, *j* and *k* are the other two main anatomical axes and A_i_, A_j_, and A_k_ are the areas under the angular velocity time-curves measured along those axes.

Finally, in order to evaluate the deviation of each specific parameter measured in patients from the reference data obtained in the control group, a Z-score was used, calculated as [18]:

$$Z_{Pi}=\{\begin{array}{ccc} \frac{\bar{P_{C}}-P_{i}}{\sigma_{C}} & for & P=RMC,NJ\\ \frac{P_{i}-\bar{P_{C}}}{\sigma_{C}} & for & P=\omega_{m},\omega_{p} \end{array};$$ (3)

where P is the parameter of interest, i is the participant and $\bar{P_{C}}$ and *σ*_*C*_ are the mean and the standard deviation values of the parameter P measured in the control group.

By summing all the scores obtained for different parameters, a composite score (Z_CS_) was calculated for each patient, as associated to the performance of a specific movement:

$$Z_{CS}{}_{i}=\sum_{P}Z_{Pi}.$$ (4)

As can be deducted from its formulation, the lowest the Z-score, the more the participant differs from the control group reference value calculated for that movement.

### Statistical Analysis

A reliability analysis was performed to check, for each movement and for each parameter, the level of agreement between the repeated tests. A two-way random interclass correlation coefficient (ICC (2, 1)) for a single measurement was used [19]. According to the literature, ICC values were interpreted as: good > 0.75, moderate 0.4–0.75, poor < 0.4 [20]. For those parameters that showed levels of agreement ranging from moderate to good, values obtained in the three repetitions of the various movements were averaged and retained for further analysis.

Normality of the data was verified for each parameter and movement using a Shapiro-Wilk test and parametric (independent t-test) or non-parametric (Mann-Whitney U-test) tests were then consistently adopted to quantify differences between the two groups. In both cases, statistical significance was set at an alpha level of 0.05. Cohen’s d was also computed and used as an indicator of the effect size. According to the interpretation scale reported in literature [21], the effect size was judged as negligible if d ≤ 0.2, small if 0.2 < d ≤ 0.5, medium if 0.5 < d ≤ 0.8 and large if d > 0.8.

Additionally, Spearman’s rank correlation coefficient (ρ) was calculated to evaluate the relationship between the Z-scores computed for each patient and his/her ALSFRS-R score. Statistical significance for the correlation between the Z-score and the ALSFRS-R score was set at an alpha level of 0.05.

## Results

All participants performed the head movements to reach the maximum amplitude. Among the 13 ALS participants, only a subgroup of 9 (5 females, 4 males, age range 45–74 years, ALSFRS-R score 29±11) was able to perform the head movements when asked to reach their maximum speed (See Table 1).

ICC values obtained for all movements, in trials performed at both maximum amplitude and maximum speed, are given in Table 2 and Table 3, respectively. ICC was moderate to good in all movements and for most parameters. In the ALS patients the only exceptions were observed for the NJ in extension from neutral position (E1) and in flexion back to neutral position (F2), when performed at maximum speed. In the controls, NJ in the extension from the neutral position (E1) showed a poor agreement, when performed at maximum speed. Those parameters were excluded from further analysis.

**Table 2:** ICC values for mean (ω_m_) and peak (ω_p_) angular velocity, normalized jerk (NJ) and ratio of movement coupling (RMC). ICC calculated for both ALS patients and controls (C) in the Extension (E), Flexion (F), Axial Rotation (AR) and Lateral Flexion (LF) movements performed at maximum amplitude. 1: movement from neutral position; 2: movement back to neutral position.

ICC			Amplitude					
	ω_m_		ω_p_		NJ		RMC	
	C	ALS	C	ALS	C	ALS	C	ALS
E1	0.68	0.57	0.81	0.61	0.41	0.54	0.78	0.78
E2	0.68	0.66	0.63	0.63	0.64	0.56	0.87	0.88
F1	0.71	0.65	0.48	0.65	0.48	0.42	0.87	0.92
F2	0.63	0.59	0.67	0.53	0.52	0.83	0.85	0.94
AR1	0.56	0.61	0.66	0.61	0.74	0.53	0.53	0.92
AR2	0.83	0.69	0.82	0.62	0.57	0.45	0.50	0.69
LF1	0.87	0.69	0.81	0.72	0.84	0.63	0.83	0.57
LF2	0.90	0.87	0.86	0.72	0.65	0.87	0.80	0.78

**Table 3:** ICC values for mean (ω_m_) and peak (ω_p_) angular velocity, normalized jerk (NJ) and ratio of movement coupling (RMC). ICC calculated for both ALS patients and controls (C) in the Extension (E), Flexion (F), Axial Rotation (AR) and Lateral Flexion (LF) movements performed at maximum speed. 1: movement from neutral position; 2: movement back to neutral position.

ICC			Speed					
	ω_m_		ω_p_		NJ		RMC	
	C	ALS	C	ALS	C	ALS	C	ALS
E1	0.69	0.55	0.67	0.79	< 0.40	< 0.40	0.44	0.90
E2	0.76	0.82	0.76	0.88	0.42	0.54	0.96	0.94
F1	0.42	0.65	0.74	0.59	0.48	0.70	0.65	0.89
F2	0.83	0.85	0.80	0.77	0.44	< 0.40	0.67	0.93
AR1	0.63	0.82	0.64	0.82	0.75	0.57	0.65	0.65
AR2	0.59	0.77	0.62	0.78	0.41	0.41	0.50	0.73
LF1	0.84	0.80	0.89	0.80	0.46	0.58	0.67	0.63
LF2	0.94	0.89	0.89	0.82	0.75	0.67	0.57	0.76

**Fig 4:**
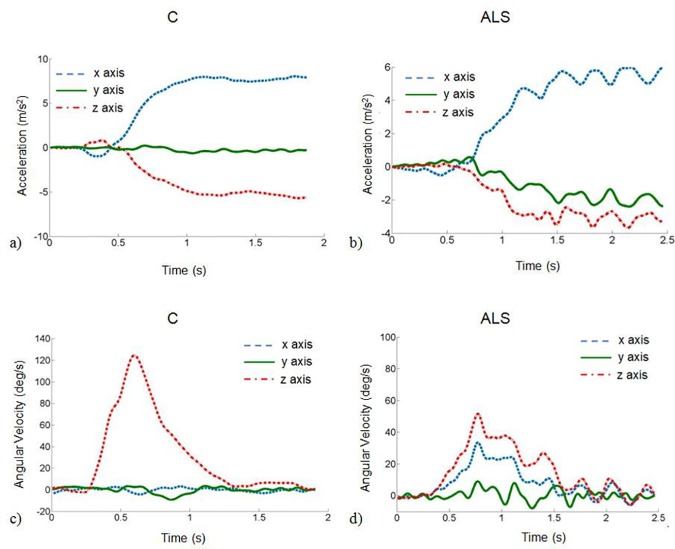
Extension from neutral position performed reaching the maximum amplitude. a) and b) Acceleration recorded when the movement was performed by the control individual (C) and the ALS patient (ALS), respectively. c) and d) Angular velocity recorded when the movement was performed by the control individual (C) and the ALS patient (ALS), respectively.

**Fig 5:**
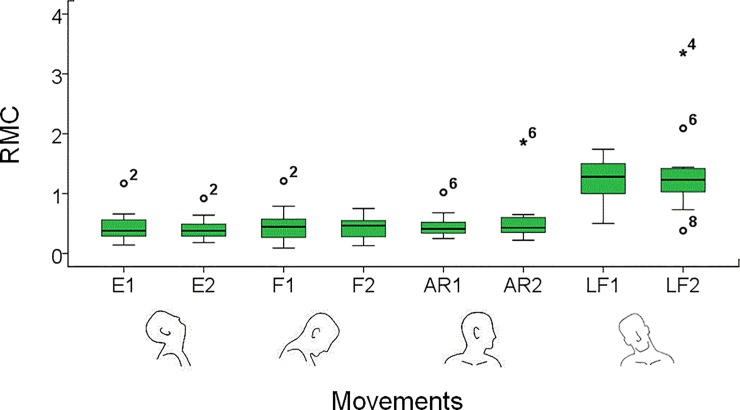
Ratio of movement coupling (RMC) values measured in movements executed by ALS patients reaching the maximum amplitude. Movements performed: Extension (E), Flexion (F), Axial Rotation (AR) and Lateral Flexion (LF). 1: movement from neutral position; 2: movement back to neutral position. Values are presented through the median, upper and lower quartiles and upper and lower extremes. Outliers and extreme outliers are represented by circles and stars, respectively. Numbers above the outliers indicate the patient associated to that value, consistently with Table 4.

A comparison between the typical signals obtained from a control individual and an ALS patient (participant Nr 3 in Table 1) is shown in Fig 4, which illustrates data from an extension of the head from the neutral position (E1), performed reaching the maximum amplitude. Table 4 shows the results obtained for both groups and for all the maximum amplitude movements. ω_m_ was significantly lower in the patient group in the extension and in the axial rotation both from (E1 and AR1, *p* = 0.012, d > 0.8 and *p* = 0.003, d > 0.8, respectively) and back (E2 and AR2, *p* = 0.010, d > 0.8 and *p* < 0.001, d > 0.8, respectively) to the neutral position and also in the lateral flexion back to neutral position (LF2, *p* = 0.009, d > 0.8). Similar results were observed in the ω_p_, where significantly lower values were measured in the ALS group, for the same movements (*p* < 0.05, d > 0.8) and for the lateral flexion from neutral position (L1, *p* = 0.048, d = 0.8). A significant reduction of movement smoothness was observed in ALS patients in the extension (E1: *p* = 0.001, d > 0.8 and E2: *p* = 0.034, d > 0.8), flexion back to neutral position (F2, *p* = 0.013, d = 0.8) and lateral flexion (LF1: *p* = 0.031, d = 0.8 and LF2: *p* = 0.016, d > 0.8) movements. A higher presence of coupled movements was observed in the ALS group in all movements (*p* < 0.05, d ≥ 0.8), except for the lateral flexion from neutral position. As highlighted in Fig 5, the inter-patient variability of the RMC values differed between movements, consistently with the variability of the pathology progression.

**Table 4:** Movements at maximum amplitude. **Mean (SD) values for mean (ω**_**m**_**) and peak (ω**_**p**_**) angular velocity, normalized jerk (NJ) and ratio of movement coupling (RMC).** Values obtained from both ALS patients and controls (C) in the Extension (E), Flexion (F), Axial Rotation (AR) and Lateral Flexion (LF) movements. 1: movement from neutral position; 2: movement back to neutral position.

Movements Max Amplitude	Mean Ang. Velocity(deg/s)		Peak Ang. Velocity(deg/s)		NJ		RMC	
	C	ALS	C	ALS	C	ALS	C	ALS
E1	31 (12)	20 (8)*	67 (27)	46 (20)*	2.1 (0.3)	3.8 (2.2)*	0.25 (0.09)	0.45 (0.26)*
E2	40 (11)	29 (10)*	75 (23)	58 (16)*	2.3 (0.5)	2.9 (0.8)*	0.24 (0.08)	0.43 (0.20)*
F1	36 (11)	34 (17)	77 (22)	73 (33)	2.5 (0.6)	3.0 (1.4)	0.23 (0.09)	0.48 (0.30)*
F2	42 (8)	34 (13)	86 (25)	69 (27)	2.1 (0.4)	3.0 (1.5)*	0.22 (0.06)	0.44 (0.19)*
AR1	49 (14)	32 (11)*	110 (33)	73 (20)*	5.1 (2.6)	5.5 (2.2)	0.26 (0.11)	0.48 (0.20)*
AR2	61 (20)	36 (10)**	109 (34)	74 (16)*	5.3 (1.8)	5.6 (2.1)	0.23 (0.09)	0.55 (0.42)*
LF1	29 (12)	23 (9)	68 (25)	51 (20)*	3.1 (1.0)	4.1 (1.4)*	1.08 (0.70)	1.22 (0.38)
LF2	40 (15)	27 (10)*	77 (28)	52 (16)*	2.6 (0.8)	3.6 (1.2)*	0.84 (0.50)	1.33 (0.73)*

(*) Level of significance for the difference between ALS and C < 0.05.

(**) Level of significance for the difference between ALS and C < 0.001.

The results concerning the trials performed reaching the maximum speed, are shown in Table 5 for both groups. The ω_m_ and ω_p_ were significantly lower (*p* < 0.05, d > 0.8) in the ALS group in extension, axial rotation and lateral flexion from the neutral position (see values in Table 5). The most remarkable difference was observed in the axial rotation from and back to the neutral position, where values measured in ALS were almost half those measured in the control group, both for the ω_m_ and the ω_p_. Movement performed by the ALS group did not show a significant reduction (*p* > 0.05) of movement smoothness in any of the movements performed, while the presence of coupled movements was significantly higher (*p* < 0.05, d > 0.8) in the flexion and in the axial rotation movements, both from and back to the neutral position.

**Table 5:** Movements at maximum speed. **Mean (SD) values for mean (ω**_**m**_**) and peak (ω**_**p**_**) angular velocity, normalized jerk (NJ), and ratio of movement coupling (RMC).** Values obtained from both ALS patients and controls (C) in the Extension (E), Flexion (F), Axial Rotation (AR) and Lateral Flexion (LF) movements. 1: movement from neutral position; 2: movement back to neutral position.

Movements Max Speed	Mean Ang. Velocity (deg/s)		Peak Ang. Velocity (deg/s)		NJ		RMC	
	C	ALS	C	ALS	C	ALS	C	ALS
E1	80 (28)	45 (12)*	163 (55)	93 (26)*	-	-	0.26 (0.11)	0.42 (0.17)
E2	87 (28)	57 (20)*	170 (58)	108 (44)*	2.3 (0.5)	2.1 (0.3)	0.24 (0.13)	0.37 (0.13)
F1	91 (16)	77 (25)	176 (35)	147 (37)	2.5 (0.4)	2.5 (0.4)	0.24 (0.09)	0.49 (0.35)*
F2	87 (36)	63 (25)	159 (59)	123 (48)	2.3 (0.5)	-	0.22 (0.06)	0.47 (0.27)*
AR1	136 (40)	73 (17)*	263 (68)	147 (27)*	3.9 (1.7)	3.4 (0.5)	0.24 (0.06)	0.45 (0.12)**
AR2	122 (30)	63 (20)**	223 (58)	121 (34)**	3.6 (0.6)	3.8 (0.9)	0.25 (0.05)	0.45 (0.11)*
LF1	64 (28)	39 (13)*	131 (53)	84 (27)*	3.2 (0.8)	3.3 (0.5)	1.05 (0.53)	1.30 (0.59)
LF2	66 (28)	44 (16)	126 (49)	86 (26)	2.8 (0.6)	3.3 (0.9)	0.91 (0.47)	1.20 (0.55)

(*) Level of significance for the difference between ALS and C < 0.05.

(**) Level of significance for the difference between ALS and C <0.001.

**Fig 6:**
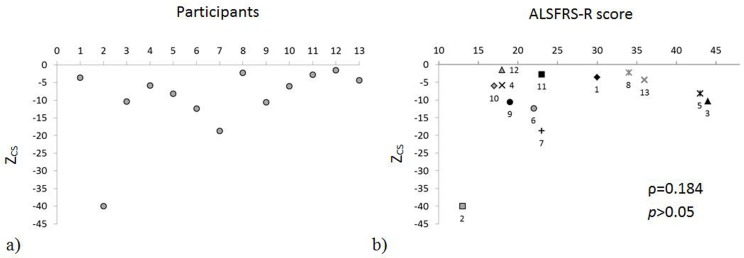
**a) Z**_**CS**_ **calculated during the extension from neutral position (E1) reaching the maximum amplitude. b) Z**_**CS**_ **calculated in E1 movement plotted against the ALSFRS-R score given to participants at the time of recording**. Numbers close to the markers indicate the participant associated to those Z and ALSFRS-R scores. ρ = Spearman’s rank correlation coefficient. Level of significance for the correlation between Z_CS_ and ALSFRS-R score: *p* < 0.05.

Fig 6A exemplifies the Z_CS_ values obtained for the E1 movement in the maximum amplitude task. Fig 6B shows the Z_CS_ obtained for each patient in E1, performed reaching the maximum amplitude, as a function of the patient’s ALSFRS-R scores. The evident absence of a correlation between the two quantities was confirmed by the non-significance (*p* = 0.548) of the Spearman correlation coefficient.

## Discussion

The aim of this pilot study was to quantitatively characterise head movements with regard to velocity, smoothness and coupling in ALS compared to aged matched controls. Despite the relatively small number of participants enrolled in this study, which represents a drawback of investigating a rare pathology and a limitation of this work, reported results demonstrated a reduced ability of patients tested to perform head movements. In particular, the observed movements in ALS, when performed at a self-selected speed, appeared to be characterized by a reduced mean and peak velocity, a reduced smoothness in a subset of movements and a higher presence of coupling movements in almost all the movements, compared to controls. In order to draw stronger conclusions on the clinical meaning of the proposed method, further studies involving a larger number of patients are needed.

Despite their limited ability, the patients managed to perform the chosen tests, which were minimally invasive for them. The level of agreement between the three repetitions performed for each movement and parameter was satisfactory overall, with ICC values ranging from moderate to high, except in a few cases. This is a very encouraging result and supports progression towards the definition of a reliable quantitative approach to overcome the current limitations of the MMT and HHD, reported in the introduction. In addition, the proposed approach might be used to measure specific neck muscle impairment, currently not provided by clinical scales such as the ALSFRS-R, which could be used in the longitudinal assessment of changes or to drive personalised intervention, such as the choice of a cervical orthosis.

Mean and peak velocities were significantly reduced in the ALS group in a subset of movements in trials at maximum amplitude and in trials at maximum speed. Those results confirmed that angular velocity is a viable parameter to identify and quantify movement impairment in ALS patients, as previously reported in patients affected by cervical dystonia [8]. The reduction of velocity is likely to be multi-factorial, relating to both muscle weakness and tone change. In addition, compensatory movement strategies developed to avoid the loss of cervical stability, using an avoidance behaviour similar to that observed in individuals affected by chronic musculoskeletal pain, may be occurring [22]. Further studies are needed to understand better the degree and relative impact of muscle weakness and tone change and to investigate these hypotheses.

The movements performed by the ALS patients, when reaching the maximum amplitude, were significantly less smooth in extension, flexion back to neutral position and lateral flexion, as illustrated by the curves presented in Fig 3A and 3B. These data highlight how the acceleration signal is more jagged when the movement is performed by a patient with ALS and confirm the presence of impaired movement coordination. The overall jerkier movements observed in patients with ALS were similar to those reported previously for other neurological conditions such as Parkinson’s disease [23] and multiple sclerosis [18]. In our ALS patients reduced coordination was, on average, only observed in a subset of movements, possibly providing an indication of the group of muscles that were more compromised in our participants. Results obtained from our participants are consistent with a significant functional deterioration of neck extensor muscles, as reported in literature for patients with ALS [2]. Our results support the hypothesis that jerkier movements could be associated with motor control strategies characterized by continuous feedback corrections, caused by an alteration of the proprioceptive input or of the feedforward control mechanisms [24]. This seems to be compatible with the nature of ALS, the major impact of which is a reduced ability to initiate and control muscle movements. Additional studies, possibly involving also upper and lower limbs movements, would help to further clarify the characteristics of motor control and movement strategies in patients with ALS and corroborate these hypotheses.

The smoothness results obtained in trials performed reaching the maximum amplitude were not confirmed in trials performed reaching the maximum speed. The presence of lower jerk values in movement performed at higher speed was consistent with experimental values found in previous studies [25]. The different results obtained could be also attributable to the different level of ability of the ALS patients that performed the faster movements (see Table 1), who were considered to be less impaired, compared to those that were able to perform only movements at maximum amplitude. An independent objective quantification of the residual patient ability, however, was not available and further studies are hence needed to verify this hypothesis.

Values obtained for the RMC in trials at maximum amplitude showed a higher presence of coupled movements when the exercises were performed by ALS patients, as can be observed by comparing two typical angular velocity signals from a control individual and an ALS patient (Fig 3C and 3D, respectively). Increased coupled movements could be caused by an alteration of central motor control and/or by the degenerative process that involves neck muscles with a consequent adoption of compensating movement strategies to maintain the orientation of the head [26]. The variability of these degenerative processes in part explains the observed RMC inter-patient variability. The absence of a significant difference between ALS patients and the control group in flexion from the neutral position is most likely due to the characteristics of the movement itself, which has been shown to be associated with an axial rotation movement also in healthy participants [27, 28]. The trials at maximum speed were performed similarly by the two groups, except for the flexion and the axial rotation movements. This may well be due to the fact that only the patients with less severe deficits managed to perform these tests. The composite Z score here proposed can be used for comparison between participants and also to quantify the (dis)similarity between the quantities measured in patients and the reference values obtained for the control group. Using this score, for example, it was possible to clearly classify patients according to their ability to perform the extension movement (E1, Fig 6A). The score proposed might be used to objectively rate and classify patients according to their ability to control their head movements and monitor relevant changes in time. Further studies, including longitudinal data from larger groups, are needed to build a larger reference dataset and validate this approach. No correlation was observed between the Z-score and the ALSFRS-R score. Although the ALSFRS-R score does correlate with overall disease progression in ALS [13] it has been shown as inadequate to accurately describe functional loss in performing arm movements [6]. The lack of correlation between the Z-score and the ALSFRS-R score may be due to a similar limitation of the ALSFRS-R to quantify functional loss in head movements. Although, the small sample size limits the possibility to draw general conclusions, a quantitative clinical scale able to detect small but potentially significant functional loss in patients with ALS may be of value.

## Conclusions

This is the first study providing a functional objective quantification of head movements in ALS. The reported results demonstrate that head movements in ALS patients, compared to age–matched controls, are characterized by reduced smoothness and velocity and by increased presence of coupling movements, which are consistent with weakness of neck extensor muscles. The ratio of movement coupling described in this study is a viable functional parameter. Further work involving different body areas and correlation with existing methods of evaluating neuromuscular function, such as dynamometry and EMG, is needed to explore the use of this approach as a marker of disease progression in ALS.

## References

[1] McDermott CJ, Shaw PJ. Diagnosis and management of motor neurone disease. British Medical Journal. 2008; 336: p. 658–662. 10.1136/bmj.39493.511759.BE 18356234 PMC2270983

[2] Gourie-Devi A, Nalini A, Sandhya S. Early or late appearance of "dropped head syndrome" in amyotrophic lateral sclerosis. Journal of Neurology, Neurosurgery, and Psychiatry. 2003; 74(5): p. 683–686. 10.1136/jnnp.74.5.683 12700323 PMC1738454

[3] Baxter S, Reed H, Clarke Z, Judge S, Heron N, Mccarthy A, et al Evaluating a novel cervical orthosis, the Sheffield Support Snood, in patients with amyotrophic lateral sclerosis/motor neuron disease with neck weakness. Amyotrophic Lateral Sclerosis and Frontotemporal Degeneration. 2016; 8421(3): p. 1–7. 10.3109/21678421.2016.1148170 26915274

[4] Nakamura R, Atsuta N, Watanabe H, Hirakawa A, Watanabe H, Ito M, et al Neck weakness is a potent prognostic factor in sporadic amyotrophic lateral sclerosis patients. Journal of Neurology, Neurosurgery, and Psychiatry. 2013;: p. 1–7. 10.1136/jnnp-2013-306020 23933739

[5] Andres PL, Skerry LM, Thornell B, Portney LG, Finison LJ, Munsat TL. A comparison of three measures of disease progression in ALS. Journal of the neurological sciences. 1996; 139 sUPPL: p. 64–70. 8899661 10.1016/0022-510x(96)00108-6

[6] Oskarsson B, Joyce NC, de Bie E, Nicorici A, Bajcsy R, Kurillo G, et al Upper extremity 3D reachable workspace assessment in ALS by Kinect sensor. Muscle & Nerve. 2016; 53(2): p. 234–241. 25965847 10.1002/mus.24703 PMC4770889

[7] Tsang SMH, Szeto GPY, Lee RYW. Movement coordination and differential kinematics of the cervical and thoracic spines in people with chronic neck pain. Clinical Biomechanics. 2013; 28: p. 610–617. 10.1016/j.clinbiomech.2013.05.009 23777907

[8] De Beyl Z, Salvia P. Neck movement speed in cervical dystonia. Movement Disorders: official journal of the Movement Disorder Society. 2009; 24(15): p. 2267–2271. 19845012 10.1002/mds.22830

[9] Yan JH, Thomas JR, Stelmach GE, Thomas KT. Developmental features of rapid aiming arm movements across the lifespan. Journal of Motor Behavior. 2000; 32(2): p. 121–140. 10.1080/00222890009601365 11005944

[10] Contreras-Vidal JL, Buch ER. Effects of Parkinson's disease on visuomotor adaptation. Experimental Brain Research. 2003; 150: p. 25–32. 10.1007/s00221-003-1403-y 12698213

[11] Feipel V, Rondelet B, Le Pallec J, Rooze M. Normal global motion of the cervical spine: an electrogoniometric study. Clinical Biomechanics. 1999; 14(7): p. 462–470. 10521629 10.1016/s0268-0033(98)90098-5

[12] Malmström E, Karlberg M, Fransson P, Melander A, Magnusson M. Primary and coupled cervical movements: the effect of age, gender, and body mass index. A 3-dimensional movement analysis of a population without symptoms of neck disorders. Spine. 2006; 31(2): p. E44–E50. 16418624 10.1097/01.brs.0000194841.83419.0b

[13] Cedarbaum JM, Stambler N, Fuller C, Hilt D, Thurmond B, Nakanishi A, et al The ALSFRS-R: a revised ALS functional rating scale that incorporates assessments of respiratory function. Neurological Sciences. 1999; 169: p. 13–21. 10.1016/s0022-510x(99)00210-5 10540002

[14] Brooks BR. El Escorial World Federation of Neurology criteria for the diagnosis of amyotrophic lateral sclerosis. Subcommittee on Motor Neuron Diseases/Amyotrophic Lateral Sclerosis of the World Federation of Neurology Research Group on Neuromuscular Diseases and th. Journal of the Neurological Sciences. 1994; 124 (Suppl 1): p. 96–107. 7807156 10.1016/0022-510x(94)90191-0

[15] Pancani S, Rowson J, Tindale W, Heron N, Langley J, McCarthy AD, et al Assessment of the Sheffield Support Snood, an innovative cervical orthosis designed for people affected by neck muscle weakness. Clinical Biomechanics. 2015; 32: p. 201–206. 10.1016/j.clinbiomech.2015.11.010 26673978

[16] Duc C, Salvia P, Lubansu A, Feipel V, Aminian K. A wearable inertial system to assess the cervical spine mobility: Comparison with an optoelectronic-based motion capture evaluation. Medical engineering & physics. 2013; 36(1): p. 49–56. 24075589 10.1016/j.medengphy.2013.09.002

[17] Tucker SW, Swartz EE, Hornor SD. Head and trunk acceleration during intermediate transport on medical utility vehicles. Clinical journal of sport medicine. 2016; 26(1): p. 53–58. 10.1097/JSM.0000000000000159 25380283

[18] Carpinella I, Cattaneo D, Ferrarin M. Quantitative assessment of upper limb motor function in Multiple Sclerosis using an instrumented Action Research Arm Test. Journal of Neuroengineering and Rehabilitation. 2014; 11(1): p. 67. 24745972 10.1186/1743-0003-11-67 PMC3998062

[19] Shrout PE, Fleiss JL. Intraclass correlations: Uses in assessing rater reliability. Psychological Bulletin. 1979; 86(2): p. 420–428. 18839484 10.1037//0033-2909.86.2.420

[20] Fleiss JL, Levin B, Cho Paik M. Statistical methods for rates and proportions 3rd edn Hoboken, NJ: John Wiley & Sons, Inc; 2003.

[21] Cohen J. Statistical power analysis for the behavioural sciences New York: Academic Press; 1977.

[22] Vlaeyen JWS, Linton SJ. Fear-avoidance and its consequences in chronic musculoskeletal pain: A state of the art. Pain. 2000; 85: p. 317–332. 10781906 10.1016/S0304-3959(99)00242-0

[23] Teulings H, Contreras-Vidal JL, Stelmach GE, Adler CH. Parkinsonism reduces coordination of fingers, wrist, and arm in fine motor control. Experimental Neurology. 1997; 146: p. 159–170. 10.1006/exnr.1997.6507 9225749

[24] Sjölander P, Michaelson P, Jaric S, Djupsjöbacka M. Sensorimotor disturbances in chronic neck pain—range of motion, peak velocity, smoothness of movement, and repositioning acuity. Manual Therapy. 2008; 13(2): p. 122–131. 10.1016/j.math.2006.10.002 17197230

[25] Vikne H, Bakke ES, Liestøl K, Sandbæk G, Vøllestad N. Muscle activity and head kinematics in unconstrained movements in subjects with chronic neck pain; cervical motor dysfunction or low exertion motor output? BMC musculoskeletal disorders. 2013; 14: p. 314 10.1186/1471-2474-14-314 24188070 PMC3840692

[26] Kent-Braun JA, Walker CH, Weiner MW, Miller RG. Functional significance of upper and lower motor neuron impairment in amyotrophic lateral sclerosis. Muscle & Nerve. 1998; 21(6): p. 762–768. 9585330 10.1002/(sici)1097-4598(199806)21:6<762::aid-mus8>3.0.co;2-5

[27] Jordan K, Jones PW, Dziedzic K. Describing three-dimensional cervical spine movement in a diseased and a non-diseased group using multilevel modelling. Statistics in Medicine. 2003; 22: p. 2365–2380. 10.1002/sim.1407 12854097

[28] Alba PD, Hons B, Sterling MM, Treleaven JM, Edwards SL, Jull GA. Cervical range of motion discriminates between asymptomatic persons and those with whiplash. Spine. 2001; 26(19): p. 2090–2094. 11698884 10.1097/00007632-200110010-00009

